# Impact of Economic Structure on the Environmental Kuznets Curve (EKC) hypothesis in India

**DOI:** 10.1186/s40008-021-00259-z

**Published:** 2021-12-18

**Authors:** Muhammed Ashiq Villanthenkodath, Mohini Gupta, Seema Saini, Malayaranjan Sahoo

**Affiliations:** 1 Department of Humanities and Social Sciences, Indian Institute of Technology Kharagpur, Kharagpur, West Bengal India; 2 Department of Humanities and Social Sciences, Jaypee Institute of Information Technology, A-10 sector-62, Noida, UP 201309 India; 3 Department of Economics Science, Indian Institute of Technology Kanpur, Kharagpur, India; 4 Department of Humanities and Social Sciences, National Institute of Technology (NIT) Rourkela, Rourkela, Odisha India

**Keywords:** CO_2_, Economic structure, EKC, India, Energy structure

## Abstract

This study aims to evaluate the impact of economic structure on the Environmental Kuznets Curve (EKC) in India. The present study deviates from the bulk of study in the literature with the incorporation of both aggregated and disaggregated measures of economic development on the environmental degradation function. For the empirical analysis, the study employed the Auto-Regressive Distributed Lag (ARDL) bounds testing approach of cointegration to analyse the long-run and short-run relationship during 1971–2014. Further, the direction of the causality is investigated through the Wald test approach. The results revealed that the conventional EKC hypothesis does not hold in India in both aggregated and disaggregated models since economic growth and its component have a U-shaped impact on the environmental quality in India. However, the effect of population on environmental quality is positive but not significant in the aggregated model. Whereas, in the disaggregated model, it is significantly affecting environmental quality. Hence, it is possible to infer that the population of the country increases, the demand for energy consumption increase tremendously, particularly consumption of fossil fuel like coal, oil, and natural gas, and is also evident from the energy structure coefficient from both models. This increase is due to the scarcity of renewable energy for meeting the needs of people. On the contrary, urbanization reduces environmental degradation, which may be due to improved living conditions in terms of efficient infrastructure and energy efficiency in the urban area leading to a negative relation between urbanization and environmental degradation.

## Introduction

The changes adopted in human activities related to the pandemic epoch of COVID-19 have led the Indian economy to a conjunction phase nearly similar to the recession period. For instance, India’s GDP contracted to 23.9 per cent^1^ in the first quarter as compared to the same quarter of the previous year. Further, it has a vivid effect on energy consumption and carbon dioxide emission as a reduction in human mobility and shutdown of industries has led to the decline in coal and oil consumption. This trend is steepened and prevailing continuous downfall in the industrial growth and overall economic performance of India. Hence, the slowdown in the industrial sector during COVID-19 may reduce atmospheric emissions. However, the present slowdown of carbon emissions is not sustainable for the long run as carbon dioxide keeps on increasing in the atmosphere due to economic activities over the period of time. Therefore, the study raises the question of whether the COVID 19 epoch could impact carbon dioxide reduction for a longer period. For answering this question, the present study attempts to analyze the impact on carbon dioxide influenced through gross domestic production (GDP), industrial sector, energy structure, population and urbanization in India.

Moreover, environmental degradation is a global concern, and it has gained importance as carbon dioxide emission is the prime emission that will affect the worldwide natural environment (MK 2020; Villanthenkodath et al. 2021). Therefore, many nations agreed to the Kyoto protocol in 1997 so as to shield nature from exploitation. Nonetheless, it is observed that carbon dioxide emission has rapidly levelled up in developing countries like India. Also, mentioning the reflection of the COVID-19 scenario in 2020 has adversely influenced the entire globe, but considering India as a developing country, the requirement to move from back to forth is a matter of concern. In theoretical literature, the Environmental Kuznets Curve (EKC) layout the opposing relation of exhibiting environment degradation and economic development. The EKC approach is pertained dominant to the pollution and growth introduced by Grossman and Krueger (1991); also, Stern and Common (2001) explain that the low industrialization will contribute to less environmental damages. However, there is no consensus regarding the eminent effect of carbon dioxide and its complex relation with economic growth. Hasanov et al. (2019) provide a cubic function form of EKC in the literature describing the monotonic rise of GDP along with carbon dioxide in Kazakhstan, which found the EKC does not hold. Unlike existing studies in the literature, the prime focus of the present study is to examine the impact of the economic structure on the environmental quality of India through the Environmental Kuznets Curve (EKC) framework.

Besides that, the proponents of economic growth encourage the reduction of environmental degradation if the economic growth is disengaged from its effect. According to the report of the Centre for Research on Energy and Clean Air (CREA 2020), India’s carbon dioxide emission has seen a drastic fall of 15% in the first quarter during the pandemic period 2020. It may be attributed to the reduction in demand for coal, oil, and gas consumption made carbon dioxide emissions fall by 30%, witnessed for the first time in the last four decades. This fall in carbon dioxide emission is mainly due to the shutdown of the industrial sector, which majorly encourages this emissions reduction, and also India targeted for 40% reduction in emission by shifting to non-fossil fuel consumption. However, India is undergoing rapid industrial development; hence understanding these changes and their related impact on carbon dioxide emission is required for the relevant policymaking.

Moreover, it is believed that the industrial sector is a nucleus part of the economic system, transforming in scale and structure with the growth of an economy, specifically in a developing country like India (Fan et al. 2003). Meanwhile, the industry sectors are the eminent emitter of carbon dioxide, and consumers also contribute by utilizing the products of carbon dioxide. The intensity of carbon dioxide may differ with the different sectors of industrial structure in a specific region Tian et al. (2014). Hereby, the industrial structure is one of the important determinants that are associated with economic growth and carbon dioxide emissions. Thus, understanding how the association between CO_2_ emissions, economic structure in terms of industrial sector value-added, and economic growth by keeping urbanization, energy structure, and population as control variables prevails to provide the particulars for implementing the policy.

In this background, best of our knowledge, the contribution of this study is that first of its kind that builds a model of the structural transformation in the context of environmental degradation to foster industrial diversity and environmental sustainability. Further, several prevailing studies consider only the aggregate component of the economy while estimating the EKC hypothesis, but this study contributes to the literature by considering both aggregate and disaggregate components of the economy in the estimation of EKC. Also, the time series study reads the impact on carbon dioxide of economic structure and economic growth undertaking EKC hypothesis India. The study uses time-series data spanning from 1971 to 2014; it is the updated series compared to other studies, and it has relatively more data points to produce reliable outcomes.

The findings of the study portray both models at the aggregate and disaggregate level, wherein the aggregate model represents the long-run relation between CO_2_ emissions and economic growth. In contrast, the disaggregate model shows a long-run relationship between industrial value-added and CO_2_ emissions in the presence of other control variables. However, both model does not hold the conventional EKC hypothesis for India. Thus, the government authority can establish a policy targeting renewable energy over and above the non-renewable energy structures.

The paper proceeds in the following sections: Sect. 2 briefs about the related literature. Section 3 represents the theoretical model, data briefing, and econometric methodology; Sect. 4 delineates the empirical analysis; last Sect. 5 includes the conclusion and policy implication.

## Literature review

In the existing literature, the relationship between economic growth and environmental quality has been amply studied. In the book “The Limits to Growth”, Meadows et al. (1972) argue that economic growth degrades environmental sustainability. Hence, to protect the environmental quality, there should be a limit to growth. In the seminal paper, Grossman and Krueger (1991) explored the environmental impact of the North American Free Trade Agreement (NAFTA) and observed that economic growth would affect the environment by *scale effect*, *composition effect*, and *technical effect*. They also find that two pollutants, i.e., smoke and SO_2_, increase with GDP at a low level of national income, but at a higher level of income, they decrease with GDP.

Similarly, Wang et al. (2016) assessed the relationship between economic growth and sulfur dioxide emissions and found that income-sulfur dioxide emissions follow a conventional environmental Kuznets curve path. Similar results were found by Panayotou (1993), Shafik (1994), Apergis and Ozturk (2015), Bilgili et al. (2016), Shahbaz et al. (2017), El Montasser et al. (2018) while estimating the EKC hypothesis. Likewise, Stern and Common (2001) investigated the relationship between economic growth and sulfur dioxide for 74 countries globally from 1960 to 1990 but did not find evidence for the conventional EKC hypothesis. Hence, they concluded that the EKC model is fundamentally misspecified, and there is an omitted variable bias. The same outcome has reached (Harbaugh et al. 2002) while taking a similar variable as the proxy measure for environmental quality. However, Dasgupta et al. (2002) doubt the universal acceptability of the EKC hypothesis. Pal and Mitra (2017) argue that there is still another turning point, even if there is evidence for the conventional EKC relationship.

By incorporating additional variables in the CO_2_ emissions model, Wang et al. (2013) examine the impact of economic growth, population, technology level, urbanization, service level, industrialization, energy consumption structure, and foreign trade on the energy-related CO_2_ emissions in Guangdong Province, China, from 1980 to 2010 using an extended STIRPAT model. Results indicate that technology level, foreign trade degree, and energy consumption structure lead to a decline in CO_2_ emissions. In a different study, Wang et al. (2017) investigate the driving factors of CO_2_ emissions from a regional perspective in China by employing the extended STIRPAT model from 1952 to 2012. The emanated results show that the impacts and influences of various factors on carbon emissions are different in the different development stages. Likewise, Ghazali and Ali (2019) studied the impact of various factors on CO_2_ in Newly Industrialized Countries (NICs) by utilizing the extended STIRPAT model from 1991 to 2013. The empirical results of the study suggest that GDP per capita, population, and CO_2_ emission intensity along with energy intensity are main contributors for CO_2_ emissions for NICs, while population carrying capacity have no significant impact on CO_2_ emission level.

There seems to be mixed evidence of the EKC hypothesis. Grossman and Krueger (1991) suggest that the environment cannot be controlled by economic growth unless supported by institutions and policies. Therefore, the EKC hypothesis's validation depends intuitively on other factors such as access to technology or technological progress, quality of institution & availability of natural resources (Dogan and Inglesi-Lotz 2020). Recent studies have also included other variables like energy consumption, foreign aid, corruption, foreign investment, urbanization, technology, energy intensity, and financial development (Mahalik et al. 2021; Villanthenkodath and Mahalik 2020). Hence, Carson (2010) points those results are sensitive to the model specification, dataset, variable added, and environmental proxy.

In the Indian context, a review of literature also shows mixed evidence of the EKC hypothesis. For instance, Boutabba (2014) examines the causal relationship's existence and direction in a multivariate framework for the Indian economy from 1970 to 2008. Their results suggest the long-run relationship between the per capita income and per capita carbon emission and further lend support to the EKC hypothesis. Similarly, Sehrawat and Giri (2015), using urbanization as an additional contributor to the emissions, attempted to study the EKC hypothesis during 1971–2011 for the Indian economy. They confirm the existence of the EKC hypothesis. Besides that, Kanjilal and Ghosh (2013), using a threshold cointegration test, found the presence of the EKC hypothesis for India. Likewise, Jayanthakumaran et al. (2012) concluded in favour of the EKC hypothesis in India. Recently, Shahbaz and Sinha (2019) estimated the EKC for emissions using the ARDL technique from 1971 to 2015 for the Indian economy. The study includes renewable energy measured by electric power consumption and its effect on environmental quality. The results suggest that EKC does exist for India. A study conducted by Dar and Asif (2017) explored energy use, financial development, and economic growth on the emissions using the ARDL model for the Indian economy. However, the study fails to establish the presence of the EKC hypothesis. A similar outcome has been reached by Alam and Adil (2019) since they conclude that there is no significant relationship between economic growth and carbon emissions. A study by Roy et al. (2017) analyzed the environmental impact of energy demand, energy mix, and fossil fuel intensity in a fast-growing economy like India from 1990 to 2016. They find that population, energy structure, and energy intensity are statistically factors for the CO_2_ emission in India.

Some studies consider the various economic growth sources to test the EKC hypothesis but do not largely exist in the literature Dogan and Inglesi-Lotz (2020) and Lin et al. (2016). Our research will bridge this gap by studying the EKC hypothesis's presence by considering different economic growth sources for the Indian economy.

## Theoretical model, data description, and econometric methodology

### Theoretical model and data description

The IPAT identity is considered as a system for determining what constitutes the patterns of the environment (Chertow 2000). The framework demonstrates how climate change (Generally calculated in terms of either CO_2_ or other air pollutants) responds to factors such as population, affluence, and technology.

1 $$I = PAT$$

In Eq. 1, $I$ stands for the degradation of environmental quality proxy in terms of emissions, $P$ measures the growth of population. $A$ is the affluence of society measured in terms of GDP, $T$ used as technology proxy.

Dietz and Rosa (1997) introduced the STIRPAT model due to the criticism related to earlier IPAT model assumptions such as the elasticities of all parameters are each equal and the simplicity (Tursun et al, 2015; Wang and Zhao 2015).

2 $$I_{t} = \alpha P_{t}^{\beta } A_{t}^{\gamma } T_{t}^{\delta } \mu_{t}$$

In Eq. 2, $\alpha$ indicates the intercept, $P$, $A,$ and $T$ follows the same meaning of Eq. 1. $\beta , \gamma$ and $\delta$ indicates the elasticities of related to the impact of $P$, $A$ and $T$ on the environment. Subscript $t$ measures the year and $\mu_{t}$ is the stochastic error term in the model.

The underpinning theoretical framework of this study was proposed by Dogan and Inglesi-Lotz (2020) and Lin et al. (2016). For evaluating the determinants of CO_2_ emissions, these studies extended the STIRPAT model. Lin et al. (2016) modified the equation of STIRPAT by incorporating the square of GDP, energy structure, and urbanization of the countries. Similarly, Dogan and Inglesi-Lotz (2020) extended the STIRPAT by introducing the square term of industrial value-added in the context of European countries. Hence, the conceptualization of affluence in the STIRPAT model in both the industrial value-added and total GDP of India to analyses their impacts on emissions of CO_2_. Moreover, in any economy, the structure of energy consumption, i.e., shares of fossil fuels in total energy consumption, is an important element that influences the levels of emissions, which in turn affects the environment (You 2011).

In the Indian context, earlier studies neglect the composition and pattern of GDP and their subsequent effects on the environment; instead, the studies focus on the aggregate GDP as a measurement of economic growth. On this line, the study has set up two models for the empirical analysis by following (Dogan and Inglesi-Lotz 2020).

Model 1: Aggregate model

3 $$\ln {\text{CO}}_{2t} = \alpha_{0} + \alpha_{1} \ln {\text{CO}}_{2t - i} + \alpha_{2} \ln {\text{GDP}}_{t} + \alpha_{3} \ln {\text{GDPSQ}}_{t} + \alpha_{4} \ln {\text{POP}}_{t} + \alpha_{5} \ln {\text{URB}}_{t} + \alpha_{6} \ln {\text{ES}}_{t} + \mu_{t}$$

Model 2: Disaggregate model

4 $$\ln {\text{CO}}_{2t} = \alpha_{0} + \alpha_{1} \ln {\text{CO}}_{2t - i} + \alpha_{2} \ln {\text{IND}}_{t} + \alpha_{3} \ln {\text{INDSQ}}_{t} + \alpha_{4} \ln {\text{POP}}_{t} + \alpha_{5} \ln {\text{URB}}_{t} + \alpha_{6} \ln {\text{ES}}_{t} + \mu_{t}$$

In Eqs. 3 and 4, $CO_{2}$ is the carbon dioxide emissions, $\Delta \ln {\text{CO}}_{2t - i}$ measures the lag form of the carbon dioxide emissions, GDP stands for economic growth, ${\text{GDPSQ}}$ is the square term of the GDP, POP represent the population, $URB$ is the urbanization, $ES$ stands for the energy structure, IND means the industrial value-added and ${\text{INDSQ}}$ analyses the square term of industrial value-added. Intercept represented by $\alpha_{0}$, while $\alpha_{1} , \ldots \alpha_{6}$ stands for the coefficients of the explanatory variables in the model. Variables of the study are represented in Table 1, which offers the definition, measurement, and source of each variable for the period of 1971–2014. The selection of years was dictated by the availability of data for all the variables, particularly the energy structure data, which is available only up to 2014 in the World Development Indicators. The data were converted into the natural logarithm for the empirical analysis by the following studies (Pal et al. 2021; Sahoo et al. 2021; Villanthenkodath and Arakkal 2020; Villanthenkodath and Mushtaq 2021; Ansari and Villanthenkodath 2021; Villanthenkodath and Mahalik 2021).

**Table 1:** Definition of variables

Variable	Definition	Measurement	Source
CO_2_	CO_2_ emissions	Metric ton	World Development Indicators
GDP	Gross domestic product	Constant of 2010 US$	World Development Indicators
URB	Urbanization	Percent	World Development Indicators
POP	Population	Percent	World Development Indicators
ES	Energy structure	Share of fossil fuels (percent)	World Development Indicators
IND	Industry, value-added	Constant of 2010 US$	World Development Indicators

Source: Authors' compilations

### Econometric methodology

#### Stationarity test

The first phase in the empirical analysis is to determine the order of integration of the variables for choosing the appropriate econometric models for the analysis. To attain this objective, we have employed the augmented Dickey-Fuller (ADF) and Phillips-Perron (PP) unit root tests. The null hypothesis of the non-stationarity is examined in opposition to the alternative hypothesis of stationarity. The first difference stationary or I (1) series indicates that all the variables are non-stationary in the levels, but it becomes stationary at their first difference. If the variables are I (0), then such variables are level stationery.

#### Cointegration analysis

The Autoregressive Distributed Lag (ARDL) bounds testing approach of cointegration proposed by Pesaran and Shin (1995) and Pesaran et al. (2001) has been employed for establishing the long-run relationship between the variables. The ARDL bounds testing approach is superior to other cointegration methods that can be listed as follows. Firstly, it can be applied in the case of a small sample size. Secondly, irrespective of the order of integration, i.e., I(0)/I(1) or mixed integration order of the variables, this method can be employed. Thirdly, the problem of endogeneity can be solved by using the optimal lag in the model specification. Fourthly, it offers superior results over other conventional cointegration.

Model 1, i.e., the aggregate model estimated using the ARDL bounds testing approach based unrestricted error correction model as follows.

5 $$\Delta {\text{lnCO}}_{2t} =\, \lambda_{0} + \mathop \sum \limits_{i = 1}^{p} \lambda_{1i} \Delta \ln {\text{CO}}_{2t - i} + \mathop \sum \limits_{i = 1}^{p} \lambda_{2i} \Delta \ln {\text{GDP}}_{t - i} + \mathop \sum \limits_{i = 1}^{p} \lambda_{3i} \ln {\text{GDPSQ}}_{t - i} + \mathop \sum \limits_{i = 1}^{p} \lambda_{4i} \Delta \ln {\text{POP}}_{t - i} + \mathop \sum \limits_{i = 1}^{p} \lambda_{5i} \Delta \ln {\text{URB}}_{t - i} + \mathop \sum \limits_{i = 1}^{p} \lambda_{6i} \Delta \ln {\text{ES}}_{t - i} + \varphi_{1} \Delta \ln {\text{CO}}_{2t - 1} + \varphi_{2} \Delta \ln {\text{GDP}}_{t - 1} + \varphi_{3} \Delta \ln {\text{GDPSQ}}_{t - 1} + \varphi_{4} \Delta \ln {\text{POP}}_{t - 1} + \varphi_{5} \Delta \ln {\text{URB}}_{t - 1} + \varphi_{6} \Delta \ln {\text{ES}}_{t - 1} + \mu_{t}$$

In Eq. 5 ∆ stands for the first difference operator, $\lambda_{0}$ represents the constant and $\mu_{t}$ is the stochastic error terms. The process of the bounds testing approach for the long-run relationship using ARDL is based on the Wald test or F test. The null hypothesis of no cointegration i.e. $H_{0} : \varphi_{1} = \varphi_{2} = \varphi_{3} = \varphi_{4} = \varphi_{5} = \varphi_{6} = 0$ is tested against the alternative hypothesis of cointegration, i.e., $H_{1} : \varphi_{1} \ne \varphi_{2} \ne \varphi_{3} \ne \varphi_{4} \ne \varphi_{5} \ne \varphi_{6} \ne 0$ in the long run. The decision related to the long-run relationship is based on the F-statistics. If the F-statistics surpasses the critical values, then we conclude the existence of a long-run relationship and vice versa. In case the estimated value falls in between the critical values, we cannot have precise conclusion about the cointegration. The long-run elasticities can also be estimated using Eq. 5. The error correction model is represented in the following equation.

6 $$\Delta {\text{lnCO}}_{2t}\, =\, \;\lambda_{0} + \mathop \sum \limits_{i = 1}^{p} \lambda_{1i} \Delta \ln {\text{CO}}_{2t - i} + \mathop \sum \limits_{i = 1}^{p} \lambda_{2i} \Delta \ln {\text{GDP}}_{t - i} + \mathop \sum \limits_{i = 1}^{p} \lambda_{3i} \ln {\text{GDPSQ}}_{t - i} + \mathop \sum \limits_{i = 1}^{p} \lambda_{4i} \Delta \ln {\text{POP}}_{t - i} + \mathop \sum \limits_{i = 1}^{p} \lambda_{5i} \Delta \ln {\text{URB}}_{t - i} + \mathop \sum \limits_{i = 1}^{p} \lambda_{6i} \Delta \ln {\text{ES}}_{t - i} + \varphi {\text{ECT}}_{t - 1} + \mu_{1}$$

In Eq. 5, $\text{ECT}$ stands for the error correction term, the coefficient of error correction term, i.e. $\varphi$ has to be negative and less than one, and it shows the time taken for the adjustment towards the long-run equilibrium. In model 2, the empirical analysis has been carried out similarly by replacing the GDP with IND and GDPSQ with INDSQ in Eqs. 5 and 6.

## Empirical results and discussion

**Fig. 1:**
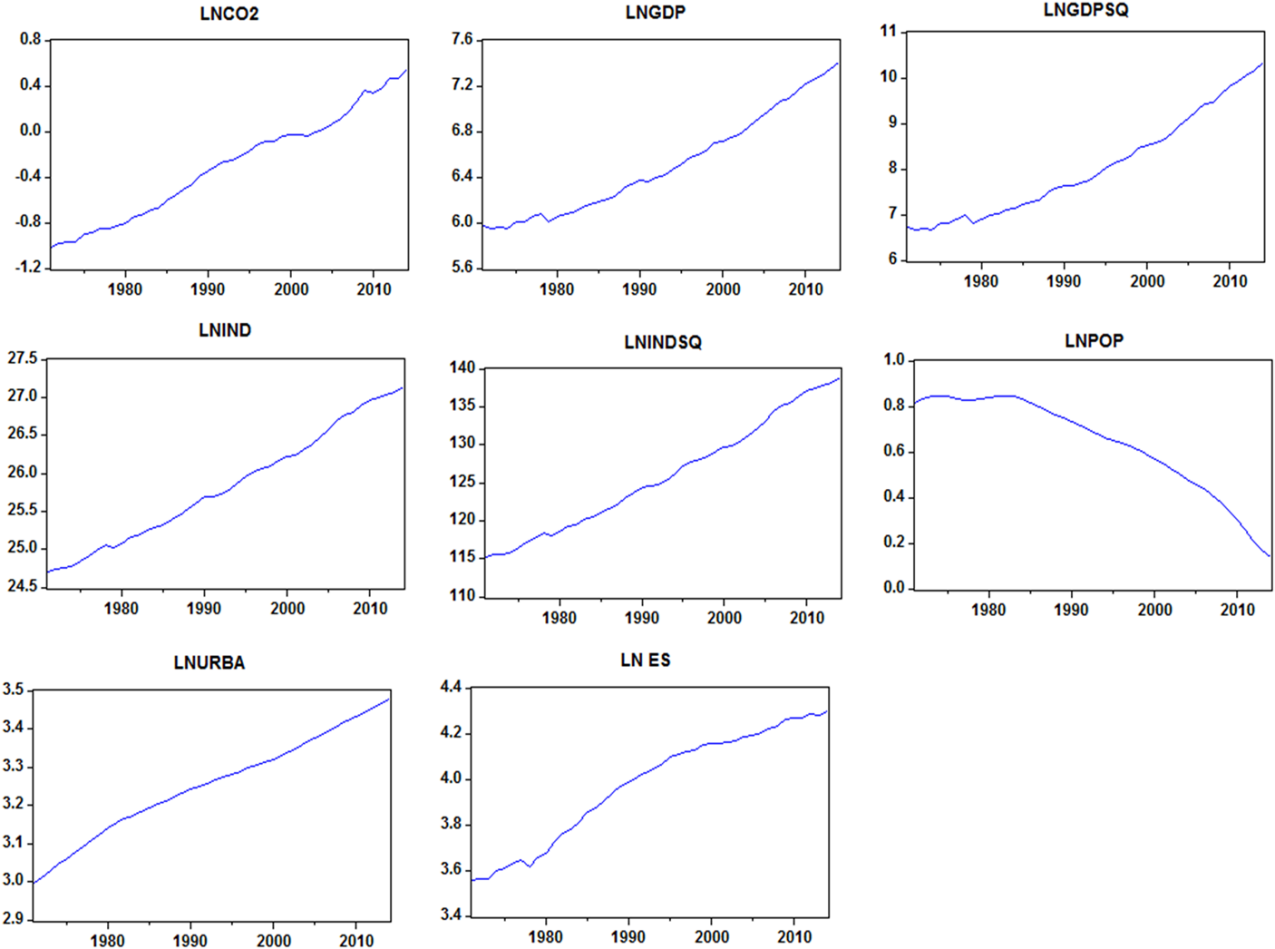
Visual plot of variables

The focus of this section is on the empirical simulations carried out in this study. First, preliminary analysis in terms of summary statistics is followed by correlation matrix analysis and then the visual plot of all variables under consideration. Table 2 highlights the descriptive statistics with industrial sector value added is having the highest average with the highest minimum and maximum, while industrial value-added, economic growth, and CO_2_ emissions are positively skewed. However, the population, urbanization, and energy sector are negatively skewed throughout the inquiry. Table 3 represents the analysis of the Pearson correlation matrix of the studied variables. The outcome shows the linear association between the variables. Moreover, there is a positive and significant relationship between CO_2_ emissions and industrial value-added. A similar conclusion has been reached for economic growth. This result indicates that industrial value-added, economic growth, urbanization, and energy structure drive environmental degradation in India. However, the population delineates a negative association with environmental degradation. Hence, to substantiate the outcomes of the correlation analysis, more analysis is needed. Figure 1 depicts the trend and pattern of the studied variables; it is clear that a positive correlation trend has been established for all the variables except population.

**Table 2:** Summary statistics

	LNCO_2_	LNGDP	LNGDPSQ	LNIND	LNINDSQ	LNPOP	LNURBA	LNES
Mean	− 0.301	6.509	8.030	25.832	125.968	0.635	3.253	3.972
Median	− 0.255	6.402	7.730	25.746	125.023	0.687	3.261	4.034
Maximum	0.544	7.403	10.336	27.140	138.925	0.847	3.478	4.298
Minimum	− 1.015	5.944	6.664	24.709	115.156	0.136	2.995	3.559
Std. Dev	0.465	0.451	1.126	0.756	7.388	0.213	0.133	0.247
Skewness	0.064	0.461	0.535	0.184	0.218	− 0.824	-0.173	− 0.385
Kurtosis	1.823	1.940	2.028	1.802	1.816	2.556	2.093	1.703
Jarque–Bera	2.568	3.615	3.827	2.879	2.917	5.336	1.728	4.172
Probability	0.277	0.164	0.148	0.237	0.233	0.069	0.421	0.124
Sum	− 13.239	286.415	353.298	1136.626	5542.611	27.924	143.150	174.787
Sum Sq. Dev	9.306	8.752	54.494	24.579	2346.825	1.943	0.764	2.617
Observations	44	44	44	44	44	44	44	44

Source: Authors' estimation

**Table 3:** Correlation matrix

	LNCO_2_	LNGDPSQ	LNGDP	LNIND	LNINDSQ	LNPOP	LNURBA	LN_ES
LNCO_2_	1							
	–							
LNGDPSQ	0.977 0.000	1						
		–						
LNGDP	0.981 0.000	1.000 0.000	1					
			–					
LNIND	0.993 0.000	0.990 0.000	0.993 0.000	1				
				–				
LNINDSQ	0.993 0.000	0.991 0.000	0.994 0.000	1.000 0.000	1			
					–			
LNPOP	− 0.951 0.000	− 0.990 0.000	− 0.986 0.000	− 0.963 0.000	− 0.966 0.000	1		
						–		
LNURBA	0.988 0.000	0.961 0.000	0.966 0.000	0.988 0.000	0.986 0.000	− 0.922 0.000	1	
							–	
LN_ES	0.981 0.000	0.927 0.000	0.937 0.000	0.968 0.000	0.965 0.000	− 0.880 0.000	0.980 0.000	1
								–

Source: Authors' estimation

In time series modelling, the need for stationary analysis is important for circumventing spurious effects. The current study has implemented the traditional unit root test ADF and PP to analyze the stationarity properties of the variables, as seen in Table 4. The outcomes of the unit root test reveal a mixed order of integration among the variable vector under review.

**Table 4:** ADF and PP and tests of unit root

Level	ADF		PP	
	Intercept	Intercept and trend	Intercept	Intercept and trend
LNCO_2_	1.058	− 1.819	1.020	− 2.012
LNGDP	3.305	− 1.830	5.396	− 1.940
LNGDPSQ	4.040	− 1.327	6.890	− 1.363
LNPOP	0.669	− 1.508	6.681	− 0.237
LNURBA	− 4.107	− 4.894*	− 4.107*	− 4.763*
LNES	− 1.694	− 0.138	− 1.503	− 0.419
LNIND	0.981	− 2.616	1.715	− 2.378
LNINSQ	1.194	− 2.369	2.031	− 2.186
First difference				
ΔLNCO_2_	− 6.121*	− 6.280*	− 6.167*	− 6.308*
ΔLNGDP	− 6.388*	− 8.280*	− 6.386*	− 14.602*
ΔLNGDPSQ	− 5.741*	− 8.158*	− 5.802	− 14.638
ΔLNPOP	− 2.942***	− 8.344*	− 2.357***	− 8.245*
ΔLNURBA	–	–	–	–
ΔLNES	− 5.446*	− 5.890*	− 5.550*	− 5.915
ΔLNIND	− 4.705*	− 4.830*	− 4.681*	− 4.890*
ΔLNINSQ	− 4.718*	− 4.987*	− 4.550*	− 4.828*

* and *** indicates 1% and 10% statistical significance

Source: Authors' estimation

Successively, the study has established the long-run relationship between the variables with the help of Pesaran’s ARDL Bounds test. The result shows the clear existence of a long-run relationship among the series that has been explored in the study. Optimum parsimonious lag has been chosen by the Akaike Information Criterion (AIC) (Table 5).

**Table 5:** ARDL bounds test

Model: 1	$\ln {\text{CO}}_{2t} = f\left( {\ln {\text{GDP}}_{t} ,\ln {\text{GDPSQ}}_{t} ,\ln {\text{POP}}_{t} , \ln {\text{URB}}_{t} ,\ln {\text{ES}}_{t} } \right)$			
Model: 2	$\ln {\text{CO}}_{2t} = f\left( {\ln {\text{IND}}_{t} ,\ln {\text{INDSQ}}_{t} ,\ln {\text{POP}}_{t} , \ln {\text{URB}}_{t} ,\ln {\text{ES}}_{t} } \right)$			
Test statistic	Value	Signif	I(0)	I(1)
F-statistic		10%	2.08	3
Model: 1	12.195	5%	2.39	3.38
Model: 2	9.327	2.50%	2.7	3.73
k	5	1%	3.06	4.15

Critical value of Narayan (2005) has used by authors

Source: Authors' estimation

The long-run and short-run result obtained from Model 1 and Model 2 is reported in Tables 6 and 7. Model 1 displays the outcomes of the model using total GDP to reflect the economic growth, whereas Model 2 employs growth of the industrial sector which is used as an affluence proxy. The obtained result shows that the conventional EKC hypothesis is not holding in both the models; rather, it shows a U-shaped relation between the affluence proxies, i.e., GDP and IND, since the sign of coefficient on GDP and IND is negative, and GDPSQ and INDSQ is positive. Our findings are in line with Alam and Adil (2019) and Dar and Asif (2017). However, it differs from Jayanthakumaran et al. (2012) and Shahbaz and Sinha (2019).

**Table 6:** ARDL results Model 1

Variable	Coefficient	Std. Error	t-Statistic	Prob
Long run				
LNGDP	− 6.866*	2.380	− 2.885	0.009
LNGDPSQ	3.040*	1.136	2.675	0.014
LNPOP	0.405	0.924	0.438	0.666
LNURBA	− 2.762*	0.983	− 2.809	0.011
LNES	2.249*	0.374	6.013	0.000
C	19.794*	7.203	2.748	0.012
Short run				
D(LNGDP)	− 4.494*	1.364	− 3.295	0.004
D(LNGDPSQ)	1.957*	0.583	3.359	0.003
D(LNPOP)	12.904**	4.860	2.655	0.015
(LNURBA)	− 1.687*	0.532	− 3.174	0.005
(LNES)	1.374*	0.227	6.064	0.000
ECT_(*t*−1)_	− 0.611*	0.058	− 10.477	0.000
Diagnostic test				
* χ*2 NORMAL	0.514	[0.773]	*R*^2^	0.84
* χ*2 SERIAL	1.618	[0.224]	Adj *R*^2^	0.77
* χ*2 RAMSEY	1.325	[0.314]	D-W test	1.64
* χ*2 ARCH	0.128	[0.723]	F-statistic	1616.219*

* and ** indicate 1% and 5% level, respectively

Source: Authors' estimation

**Table 7:** ARDL results Model 2

Variable	Coefficient	Std. Error	*t*-Statistic	Prob
Long run				
LNIND	− 20.758*	4.150	− 5.003	0.000
LNINDSQ	2.205*	0.428	5.146	0.000
LNPOP	2.011*	0.503	4.002	0.000
LNURBA	− 1.718*	0.495	− 3.468	0.002
LN_ES	2.408*	0.322	7.471	0.000
C	253.034	52.043	4.862	0.000
Short-run				
D(LNIND)	10.020*	3.275	3.060	0.005
D(LNINDSQ)	− 1.078*	0.342	− 3.149	0.004
D(LNPOP)	10.077*	1.446	6.968	0.000
(LNURBA)	− 1.135*	0.411	− 2.759	0.010
(LN_ES)	1.591*	0.249	6.381	0.000
ECT_(*t*−1)_	− 0.661	0.074	− 8.877	0.000
Diagnostic test				
* χ*^2^ NORMAL	0.422	[0.809]	*R*^2^	0.71
* χ*^2^ SERIAL	0.022	[0.978]	Adj *R*^2^	0.66
* χ*^2^ RAMSEY	0.935	[0.358]	D-W test	1.97
* χ*^2^ ARCH	0.020	[0.889]	F-statistic	2055.322*

* indicate 1% statistical significance level

Source: Authors' estimation

In line with the preconceived notion, if other things remain constant, then the population growth coefficient has a positive effect on increasing levels of emission in both the short-run and long-run across the models. In model 1, the long-run coefficient is not significant when aggregate GDP is used. However, the short-run coefficient is positive and significant. In model 2, the population has a positive and significant impact on pollution both in the short-run and long-run when disaggregate GDP is employed. It may be due to an increase in the population contributing to the rising need for energy consumption. Similarly, the demand for goods and services also spur population growth; hence the energy required to produce the consumption goods also increases, which in turn enhances the CO_2_ emissions. In the literature, Song et al. (2015) and Gertler et al. (2013) observed that population growth could also be complemented by an increase in general economic conditions, living standards, and household income levels; as a result, there is a rise in energy consumption and emissions of CO_2_.

In both models, the level of urbanization is negative and statistically significant in the short-run and long run. It indicates that urbanization has historically created a positive effect on environmental degradation, especially at the early stages of urbanization. However, the improved living conditions in terms of efficient infrastructure and energy in an urban area lead to a negative relation between urbanization and environmental degradation. The accelerating force behind such a move may be that replacing the inefficient energy sources with more efficient energy sources. This finding is consistent with the studies that found the negative relationship between urbanization and emissions of CO_2_ (Pachauri 2004; Poumanyvong and Kaneko 2010; Burton 2000; Pachauri and Jiang 2008).

In both models, the energy structure coefficient is positive and significant in the long run and short run. Therefore, the study concluded that the composition of fossil fuels in the mix of energy is a driver of CO_2_ emissions. These findings support the theory that fossil fuels use is the major contributor to the increase in emissions. Therefore, our findings agree with previous studies MK (2020) for India and Canadell et al. (2009) for South Africa.

**Fig. 2:**
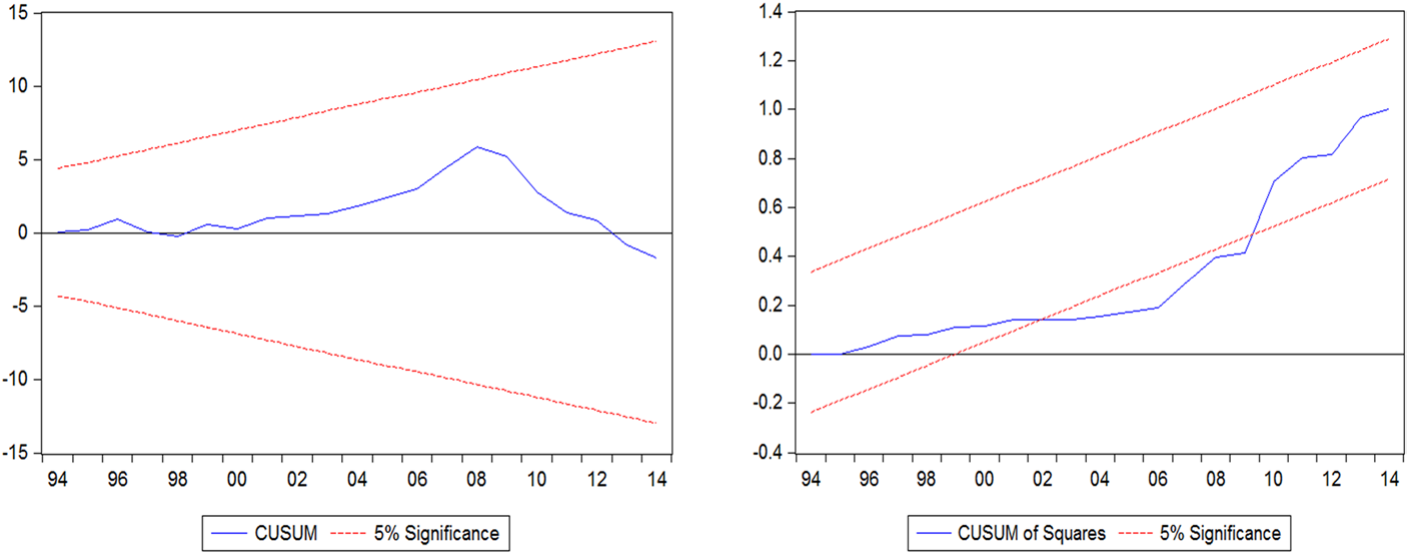
CUSUM and CUSUMsq for Model 1

**Fig. 3:**
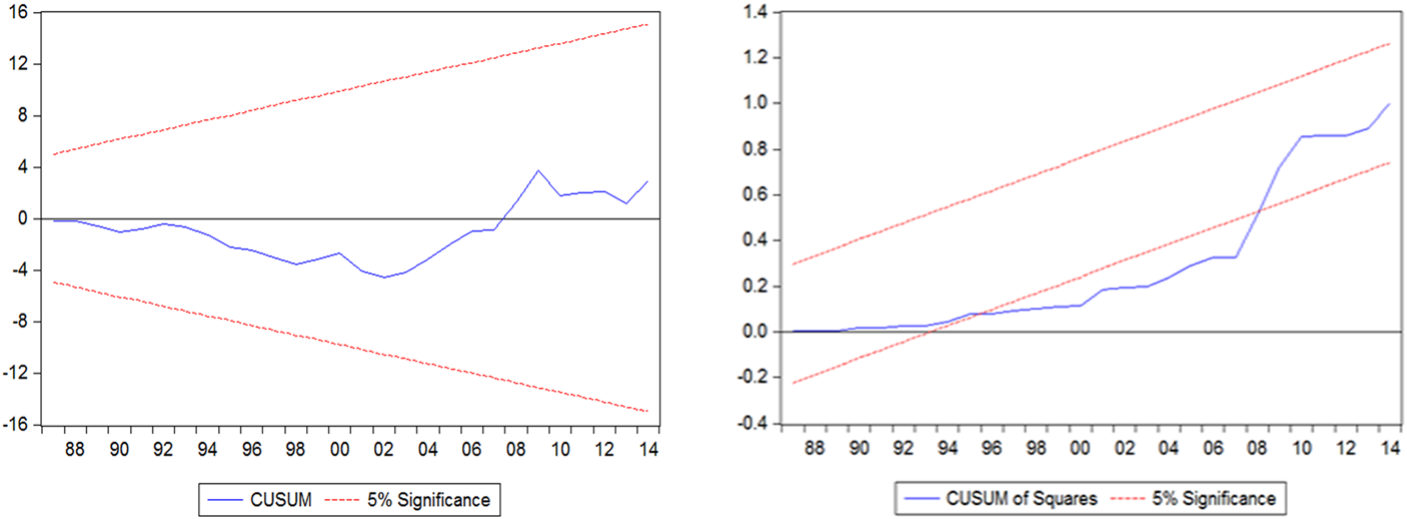
CUSUM and CUSUMsq for Model 2

The incorporated error correction term in both models shows that high speed in convergence to long-run equilibrium. The diagnostic test results show that both models are free from heteroscedasticity, serial correlation, and ARCH problems. ARDL models are well specified since Ramsey reset test offers the desired result. The cumulative sum of recursive residuals (CUSUM) and the CUSUM Square of recursive residuals (CUSUMsq) has been employed for both the models as proposed by Brown et al. (1975). The plot of the same is in Figs. 2 and 3.

Table 8 delineates the causality result based on the modified Wald test and corroborates the fossil fuel-induced growth hypothesis since there is a one-way causality running from energy structure (fossil fuel composition) to economic growth in India. The finding suggests that in the case of India, the fossil fuel conservation policy has to be enforced with caution; otherwise, it damages economic growth.

**Table 8:** Granger causality analysis

Excluded	Chi-sq	d*f*	Prob
Dependent variable: LNCO_2_			
LNGDP	0.271	1	0.603
LNPOP	2.085	1	0.149
LNURBA	5.329	1	0.021**
LNES	2.194	1	0.139
All	9.260	4	0.055**
Dependent variable: LNGDP			
LNCO_2_	4.224	1	0.040**
LNPOP	8.488	1	0.004*
LNURBA	4.863	1	0.027**
LNES	5.444	1	0.020**
All	14.474	4	0.006*
Dependent variable: LNPOP			
LNCO_2_	1.436	1	0.231
LNGDP	0.646	1	0.422
LNURBA	1.183	1	0.277
LNES	1.588	1	0.208
All	23.496	4	0.000*
Dependent variable: LNURBA			
LNCO_2_	4.962	1	0.026**
LNGDP	4.634	1	0.031**
LNPOP	2.889	1	0.089***
LN_ES	0.627	1	0.429
All	180.119	4	0.000*
Dependent variable: LNES			
LNCO_2_	0.078	1	0.780
LNGDP	0.065	1	0.800
LNPOP	0.595	1	0.441
LNURBA	9.630	1	0.002*
All	17.707	4	0.001*

*, ** and *** indicates 1%, 5% and 10% level of significance

Authors' estimation

## Conclusion and policy implications

In this study, we are trying to examine the aggregate and disaggregate measure of economic growth and its effect on the environmental quality in India from 1971 to 2014. We have run two models to analyze the EKC hypothesis in the aggregated model, and the other one is a disaggregated model. For analyzing the long run and short run, we have applied Auto-Regressive Distributed Lag (ARDL) bound testing approach. The direction of the variables is measured through the modified Wald test.

The results revealed that the EKC hypothesis doesn’t hold in India in both aggregated and disaggregated models. In the aggregate model, considering the economic growth shows a U- relation with environmental degradation in India. In the disaggregated model, employing industrial sector value added instead of economic growth also produced a similar outcome. However, the effect of population on environmental quality is positive but not significant in model 1 or the aggregated model. Whereas, in model 2, it is significantly affecting environmental quality. As per the World Bank (2019), India is the second-highest populace country in the world after China, and it has forecasted that it may cross the China population by 2035. So, when the population of the country increases, the demand for energy consumption increase tremendously, particularly the consumption of fossil fuel like coal, oil, and natural gas. This increases due to the scarcity of renewable energy for meeting the needs of people. Hence government should increase more investment in the renewable energy sector (solar and wind energy etc.) to increase the environmental quality in India. In this regard, foreign direct investment needs to be attracted to boost the performance of renewable energy in India. Moreover, renewable energy investment can be promoted by charging a higher price for fossil fuels or removing fossil-fuel subsidies. As a result, the demand for renewable energy probably enhances to attract new investment. In contradiction, urbanization in both models 1 and 2 shows a negative impact on the environmental quality or CO_2_ emissions. Especially at the early stages of urbanization, it has historically had a positive impact on environmental degradation. “Urbanization helps more residents to gain connections at competitive rates to environment-friendly infrastructure and services”. Innovation, like renewable technology, is driven by urbanization. In the long run, the future of the green economy can be decided by environmentally friendly facilities, machinery, cars, and services.

From the above findings, as the primary drivers of CO_2_ emissions, the chemical and heavy industries play a crucial role in India. This country ought, however, to intensify the structural transformation. Administrative means of these sectors and the promotion of low and light emission industries by fostering industrial diversity. Besides, people should increase their eco-friendly knowledge and waste recycling to reduce emissions. The better health condition of an urban resident can be achieved by stringent environmental policy and environmental awareness among the urban as well as the general population of the country. The result indicates that the policy of conservation of fossil fuels must be pursued with precaution in the case of India; otherwise, it hurts economic development.

## Data Availability

The datasets used and/or analyzed during the current study are available from the corresponding author on reasonable request.

## Footnotes

1 As reported by Industry body FICCI's Economic Outlook Survey (http://www.ficci.in/ficci-surveys.asp).
